# Metabolite changes by combined treatment, ethyl formate and low temperature, in *Drosophila suzukii*

**DOI:** 10.1038/s41598-024-77436-0

**Published:** 2024-10-29

**Authors:** Junbeom Lee, Hyun-Kyung Kim, Jong-Chan Jeon, Seung-Ju Seok, Gil-Hah Kim, Hyun-Na Koo, Dae-Weon Lee

**Affiliations:** 1 Metabolomics Research Center for Functional Materials, Kyungsung University, Busan, 48434 Republic of Korea; 2 Department of Plant Medicine, College of Agriculture, Life and Environment Science, Chungbuk National University, Cheongju, 28644 Republic of Korea; 3 Department of SmartBio, Kyungsung University, Busan, 48434 Republic of Korea; 4 Department of Plant Medicine, Chungbuk National University, Cheongju, 28644 Republic of Korea

**Keywords:** *Drosophila suzukii*, Ethyl formate, Low temperature, Metabolomics, Sphingolipids, Entomology, Animal physiology

## Abstract

Although ethyl formate (EF) fumigant and low temperature applications are widely used for pest management, studies related to their mechanisms of action and subsequent metabolic changes in *Drosophila suzukii* models are still unclear. In this study, a comparative metabolome analysis was performed to investigate the major metabolites modified by EF and low temperature and how they are related to and affect insect physiology. Most of the identified metabolites function in metabolic pathways related to the biosynthesis of amino acids, nucleotides and cofactors. In addition, a combined treatment with EF and low temperature significantly altered the tricarboxylic acid cycle (TCA) and the levels of the purine and pyrimidine classes of metabolites. Interestingly, the levels of cytochrome P450 and glutathione metabolites involved in detoxification dramatically changed under stress conditions compared to those in the control group.

**Supplementary Information:**

The online version contains supplementary material available at 10.1038/s41598-024-77436-0.

## Introduction

The spotted-wing fruit fly *Drosophila suzukii*(Matsumara) is a widespread insect pest that lays eggs on fresh mature fruits^1,2^. The hatched larvae burrow into the fruit, making it difficult to detect infection at an early stage, and wounds on the fruit caused by female ovipositors and larvae often result in secondary infection by pathogens^3–5^. Physical approaches involving cold treatment and pest management using fumigant pesticides are generally preferred for *D. suzukii*control^6^.

Ethyl formate (EF) is a plant volatile substance, and its insecticidal effect increases depending on its amount relative to that of carbon dioxide^7,8^. EF has a short exposure time and leaves no residue^1,9^, but it has a low penetration effect and requires a high concentration for insecticidal activity^10,11^. Additionally, abiotic management of pests using low temperatures is effectively utilized during the import and export processes as part of a quarantine step for flies like the Caribbean fruit fly *Anastrepha suspensa* and the oriental fruit fly *Bactrocera dorsalis*^2,12–14^. Since low-temperature treatment is performed simultaneously with customary low-temperature storage to maintain the commercial value of fruits and vegetables, it has the advantages of time efficiency, little product damage, simple treatment, and easy control of conditions. Recent studies have shown that a combination of physical cold treatment and chemical fumigants has a synergistic effect on increasing insecticidal activity^1,2,15^. However, it is unclear which molecules are involved in insecticidal activity under combined treatment conditions. To obtain evidence for synergistic effects in pest management, the molecular changes induced by a combination of cold conditions with fumigant EF were investigated through comparative metabolic profiling. These results demonstrate that specific metabolic pathways are correlated with combined treatment by low temperature and EF, thus expanding our understanding of complex synergistic mechanisms in pest management.

## Materials and methods

### Insect rearing

The spotted-wing fruit fly *D. suzukii*was reared in an insect room at 20 ± 1 °C and 60 ± 10% relative humidity (RH) under a photoperiod of 16 h of light and 8 h of darkness, as previously described^16,17^. The pupae and adult insects were maintained in a clean breeding dish (100 mm i.d.) supplied with artificial food and distilled water supplemented with 20% sugar.

### Preparation of the ethyl formate fumigant

EF (97.5%) was purchased from Sigma‒Aldrich (St. Louis MO, USA) and supplied by Safefume Co., Ltd. (Fumate™, 99%; Hoengseong, Republic of Korea).

### EF and thermal treatment

One hundred pupae were set on filter paper soaked in water and placed in a Petri dish. The methods used for fumigation alone, cold alone, and combined treatment were as follows^1^. (1) The fumigation effects of EF (LCT_50_, 20 mg/L) were tested in a 12 L desiccator (Bibby Scientific, Staffordshire, UK) sealed with glass stoppers at 20 ± 1 °C for 4 h. (2) Cold treatment was conducted at 1 °C for 24 h. (3) Following fumigation treatment for 4 h, the pupae were exposed to cold (1 °C) for 24 h. Mock was used as an untreated negative control for the experimental group EF and cold alone, and EF + RT (left at room temperature after fumigant treatment) was used for combined treatment. The pupae from each group were transferred to glass vials and rapidly cooled again with liquid nitrogen to stop subsequent metabolome changes^18,19^. All treatments and controls were replicated three times.

### Metabolite extraction

Total metabolites were extracted from the whole bodies of *D. suzukii*pupae in triplicate (100 insects/replicate)^17^. Briefly, each sample was suspended in 1 mL of solution (3:3:2, acetonitrile/isopropyl alcohol/water, *v*/*v*/*v*) and homogenized using a Taco Prep bead beater (Taco, Taichung, Taiwan) by turning the beater on and off at 30 s intervals for 5 min. Samples were then incubated at room temperature for 20 min and centrifuged at 2500 × *g* for 5 min at 4 °C. The supernatant was transferred to a new tube and dried under pure N_2_ gas. Dried samples were then suspended in 200 µL of 50% acetonitrile and sonicated for 5 min. Resulting supernatants were filtered with 0.22 μm pore size filters (Ultrafree-MC, Millipore, USA) and immediately loaded into the liquid chromatography-quadrupole time-of-flight tandem mass spectrometry (LC-QTOF/MS) instrument for metabolite analyses. Metabolite recovery rates for samples were investigated using internal standards (L-alanine, Sigma‒Aldrich), with this extraction process showing recovery rates of 50% or greater.

### Lipid extraction

Total lipids were extracted from the whole bodies of *D. suzukii*pupae in triplicate (100 insects/replicate) by the modified Bligh and Dyer method as described in a previous study^17,20^. Briefly, each sample was suspended in 3 mL of solution (2:1, methanol/chloroform, *v*/*v*) and homogenized using glass beads and a bead beater while turning the beater on and off at 30 s intervals for 5 min. Samples were incubated at room temperature for 20 min and centrifuged at 1750 × *g* for 10 min at 4 °C. Supernatants were transferred to new tubes to remove tissue debris. One milliliter of chloroform and 1.8 mL of water were added to each sample, followed by vortexing for 1 min. The lower layer was separated by centrifugation at 1750 × *g* for 10 min at 4 °C, followed by transfer to a new tube and drying under pure N_2_ gas. Dried samples were then suspended in 200 µL of solution (1:1, methanol/chloroform, *v*/*v*) and sonicated for 5 min. Resulting supernatants were filtered through 0.22 μm pore size filters and immediately loaded into the LC-QTOF/MS instrument for lipid analyses. Lipid recovery rates for samples were investigated using lipid standards (SPLASH^®^ LIPIDOMIX^®^ Mass Spec Standard, Avanti Polar Lipids, UK), with this extraction process showing recovery rates of 50% or greater.

### LC‒MS/MS

LC-QTOF/MS analysis was performed on a liquid chromatograph triple-quadrupole mass spectrometer (Agilent Technologies 1260 and 6530 System, Agilent Technologies, USA; Metabolomics Research Center for Functional Materials, Kyungsung University) with an electrospray ionization (ESI) source^17^. For metabolomic analysis, 5 µL of each sample was injected onto a ZORBAX Eclipse XDB-C18 column (4.6 mm × 50 mm, 1.8 μm; Agilent Technologies, USA) at 55 °C, while for lipid analysis, XSelect CSH C18 columns (4.6 mm × 100 mm, 3.5 μm; Waters, USA) were used. In the binary mobile phase system, mobile phase A was water with 0.1% formic acid, and mobile phase B was acetonitrile with 0.1% formic acid. The mobile phase with the flow rate set at 0.5 mL/min comprised the following composition conditions: initiation at 2% B followed by a linear gradient to 2% B over 1 min, 100% B at 8 min, 100% B at 10 min, 2% B at 11 min and 2% B at 20 min. Mass spectral analysis was performed using the ESI source in positive and negative modes. The capillary voltage was set to 2.0 kV in positive mode and 1.0 kV in negative mode. Metabolites with a mass range of *m*/*z* 100 to 1,000 were detected under the conditions for QTOF.

### Data processing

The data were analyzed in one batch to ensure that the parameters applied equally to all samples and were normalized using total ion intensity. All samples were annotated, filtered, scaled, and integrated using Mass Profiler Professional software (ver 14.0, Agilent Technologies, USA), and statistical analyses such as principal component analysis (PCA), Pearson correlation analysis, and Venn diagrams were conducted. Differentially up- or downregulated metabolites were compared to a control group and were defined as changes in entities with values of [raw fold change (FC)] > 1 and *P* < 0.01. Metabolite and lipid data were evaluated using MetaboAnalyst 6.0 (https://www.metaboanalyst.ca), and relevant pathways were visualized with the Kyoto Encyclopedia of Genes and Genomes (KEGG) database.

## Results and discussion

### Metabolome changes under the fumigant, low temperature and combined treatments

**Fig. 1:**
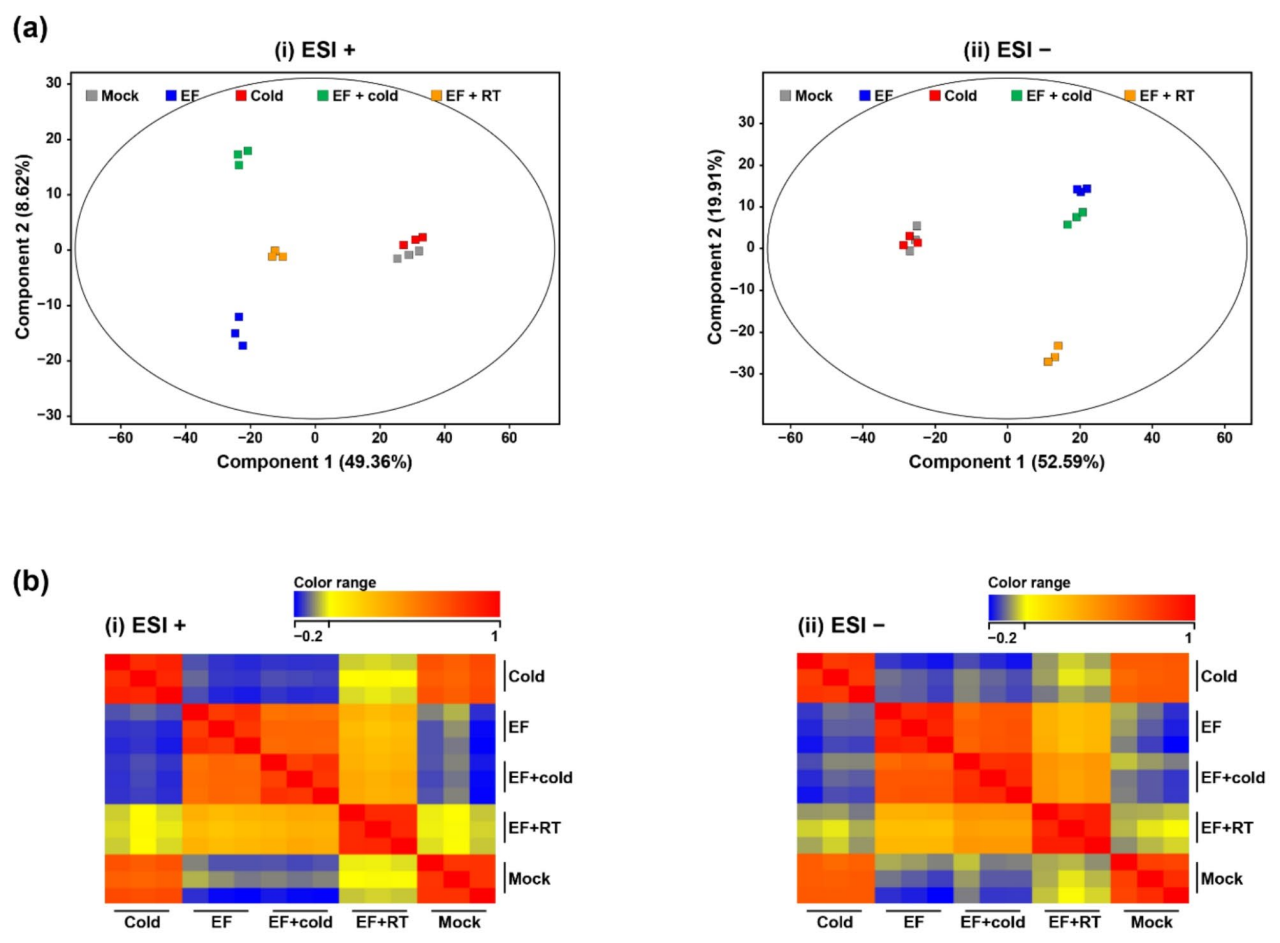
Comparative analysis of metabolome expression patterns in *D. suzukii* under different stresses.  ( a ) PCA and ( b ) correlation plots between experimental groups with altered metabolites in (i) positive (ESI+) and (ii) negative ion mode (ESI−). Each colored dot indicates the number of repetitions ( n  = 3).

Metabolomic analysis was performed to compare the physiological and biochemical changes in *D. suzukii* following combined treatment with a fumigant EF and low-temperature management. A total of 1164 and 900 metabolites were significantly detected in the positive and negative ion modes, respectively (Supplementary Data 1). Additionally, 777 and 795 metabolites were filtered through the annotation process based on the metabolite database. To investigate the reliability of these metabolome data, PCA was performed using the raw FC dataset without threshold restrictions (Fig. 1a). PCA showed well-aligned metabolic data clusters for each group and triplicate and exhibited significant distribution patterns in positive (Fig. 1a-i) and negative (Fig. 1a-ii) ion modes. These results suggest that metabolome changes in *D. suzukii* were clearly revealed by the fumigant alone, the cold alone and the combined treatments. Correlation analysis was additionally performed to reveal associations between each experimental group (Fig. 1b). Low temperature was strongly negatively correlated with EF alone and with combined treatment. These results suggest that treatment-specific indicators can be selected based on cold-specific modified metabolites via comparative metabolome analysis. The correlation analyses showed the same pattern in the positive (Fig. 1b-i) and negative (Fig. 1b-ii) ion modes.

### Distribution of metabolites according to stress conditions

**Fig. 2:**
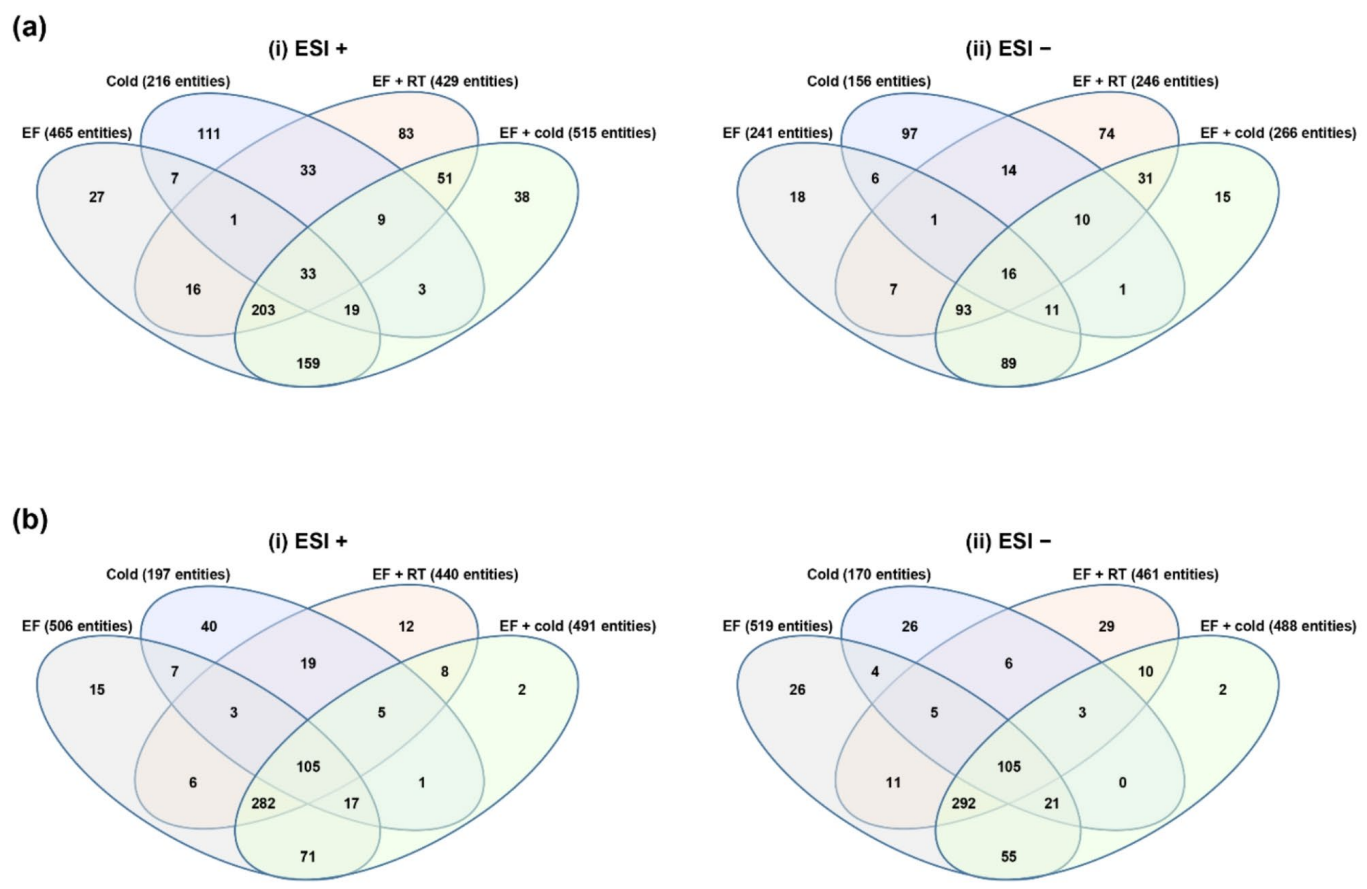
Distribution patterns of altered metabolites in *D. suzukii*.  (**a**) Upregulated and (**b**) downregulated metabolites in (i) positive and (ii) negative ion modes. Venn diagrams were constructed based on a total of 1164 positive ion mode metabolites and 900 negative ion mode metabolites. The list of metabolites is presented in Supplementary Data 1.

Venn diagram analysis was performed to determine the relationships among the metabolites that were differentially expressed under the EF, low temperature and combined treatments (Fig. 2). When the metabolites derived from each group were analyzed, 1731 metabolites were detected in the EF, 739 in the low temperature, and 1760 in the combined treatment. After annotation and filtering, 11, 5, and 23 metabolites were differentially expressed in the EF, low temperature, and combined treatment, respectively (Table 1).

**Table 1:** Metabolites specifically found under each stress condition.

	Compound	FC (raw)	Regulation	Mass
EF	PS(18:2(9Z,12Z)/0:0)	28071.42	Up	521.273
	Maltotriitol	20164.91	Up	552.188
	Oxalosuccinic acid	3394.01	Up	236.017
	Fumaric acid	111.89	Up	116.009
	O-Benzyl-L-Serine	3.36	Up	195.089
	Adenosine-3’-monophosphate (3′-AMP)	2.87	Up	347.062
	PE(14:0/0:0)	−4.23	Down	425.254
	LysoPE(0:0/16:1(9Z))	−5.27	Down	451.270
	12-Hydroxy-10-octadecynoic acid	−1705.06	Down	296.234
	2-Amino-3-methyl-1-butanol (Valinol)	−8733.85	Down	103.099
	L-Glyceric acid	−397137.60	Down	106.026
Cold	Salicin 6-phosphate	60224.79	Up	366.070
	PG(22:2(13Z,16Z)/18:0)	40047.32	Up	830.604
	3-Oxododecanoic acid	21482.78	Up	214.157
	9E,12Z,15Z-octadecatrienoic acid	1536.15	Up	278.224
	Sphinganine	2.15	Up	301.297
EF + cold	Pterin-6-carboxylic Acid	70384.41	Up	207.039
	Deoxycytidine	52196.35	Up	273.096
	L-beta-aspartyl-L-phenylalanine	8294.20	Up	280.106
	Glycyl-L-leucine	4047.69	Up	188.115
	Obliquine	1575.47	Up	448.201
	PS(19:0/0:0)	1167.58	Up	561.306
	16,16-Dimethyl Prostaglandin E2 p-(p-acetamidobenzamido) phenyl ester	757.84	Up	632.348
	UDP-N-acetyl-D-galactosamine	577.12	Up	653.086
	3,3’-Diindolylmethane	159.09	Up	246.115
	2-Furoic acid	58.55	Up	112.016
	5’-S-Methyl-5’-thioinosine	55.01	Up	358.094
	PS(22:1(11Z)/0:0)	53.29	Up	579.353
	N2-Acetyl-L-aminoadipate	52.87	Up	203.078
	Taurochenodeoxycholic acid	48.43	Up	499.294
	PA(20:0/0:0)	39.92	Up	466.306
	3’,5’-Cyclic Inosine monophosphate (cIMP)	38.42	Up	330.035
	LysoPE(0:0/18:2(9Z,12Z))	32.86	Up	499.267
	N-Palmitoyl Glycine	25.43	Up	313.261
	Indole-3-ethanol	−831.95	Down	161.084
	Hexacosanoyl-CoA	−928.97	Down	1145.505
	2-Formaminobenzoylacetate	−2392.48	Down	207.053
	L-Glutamic acid n-butyl ester	−19767.52	Down	203.115
	Uric acid	−16684734.00	Down	168.027

Oxalosuccinic acid and fumaric acid, which are intermediates in the tricarboxylic acid (TCA) cycle, were found to be specifically upregulated in the EF (Table 1). In addition, the upregulation of adenosine-3’-monophosphate (3’-AMP) is associated with the conversion of adenosine triphosphate (ATP) generated in the TCA cycle. EF functions to inhibit cytochrome c oxidase, the last enzyme in the respiratory electron transport chain (ETC) located in cell membranes^21^. In general, the TCA cycle is closely coordinated with the ETC^22^. Briefly, through a series of enzymatic reactions, the TCA cycle generates the reducing equivalents nicotinamide adenine dinucleotide (NADH) and flavin adenine dinucleotide (FADH2), which are required to transfer electrons to the ETC^22^. Considering the function of EF, cytochrome c oxidase inhibition by EF may lead to the upregulation of TCA intermediates with persistent accumulation of ATP. However, recent studies have shown that mitochondrial cytochrome c oxidase can be inhibited by ATP at high ATP/ADP ratios^23,24^. These results suggest that further studies on the mechanism of EF fumigation are needed because ATP is important for cytochrome c oxidase inhibition.

Similarly, in our study, low temperature affected glycolysis, which produces both pyruvate for the TCA cycle and some reducing power (Table 1). Salicin 6-phosphate is an intermediate product in the process by which salicin is converted into glucose 6-phosphate (KEGG PATHWAY: dme00010). The upregulation of glycolysis suggests that energy metabolism is induced to adapt to or resist cold stress. These results support previous reports that nutrient flow, metabolic plasticity and turnover increase during cold stress in the *Drosophila melanogaster*model^25^.

In addition, recent reports suggest that cuticle composition is closely related to environmental temperature, as supported by the upregulation of pterin-6-carboxylic acid in response to the combined treatment in our study (Table 1). Cold stress upregulated genes involved in chitin metabolism and cuticle binding^18^. The thermal melanism hypothesis, which states that darker-colored individuals heat faster and reach higher temperatures than lighter-colored individuals, has been suggested in Drosophilids^26^.

These results suggest that up- or downregulated metabolites independently detected in each stress treatment can also be used as indicator candidates for the corresponding treatment.

### Enrichment analysis based on metabolite profiling

**Fig. 3:**
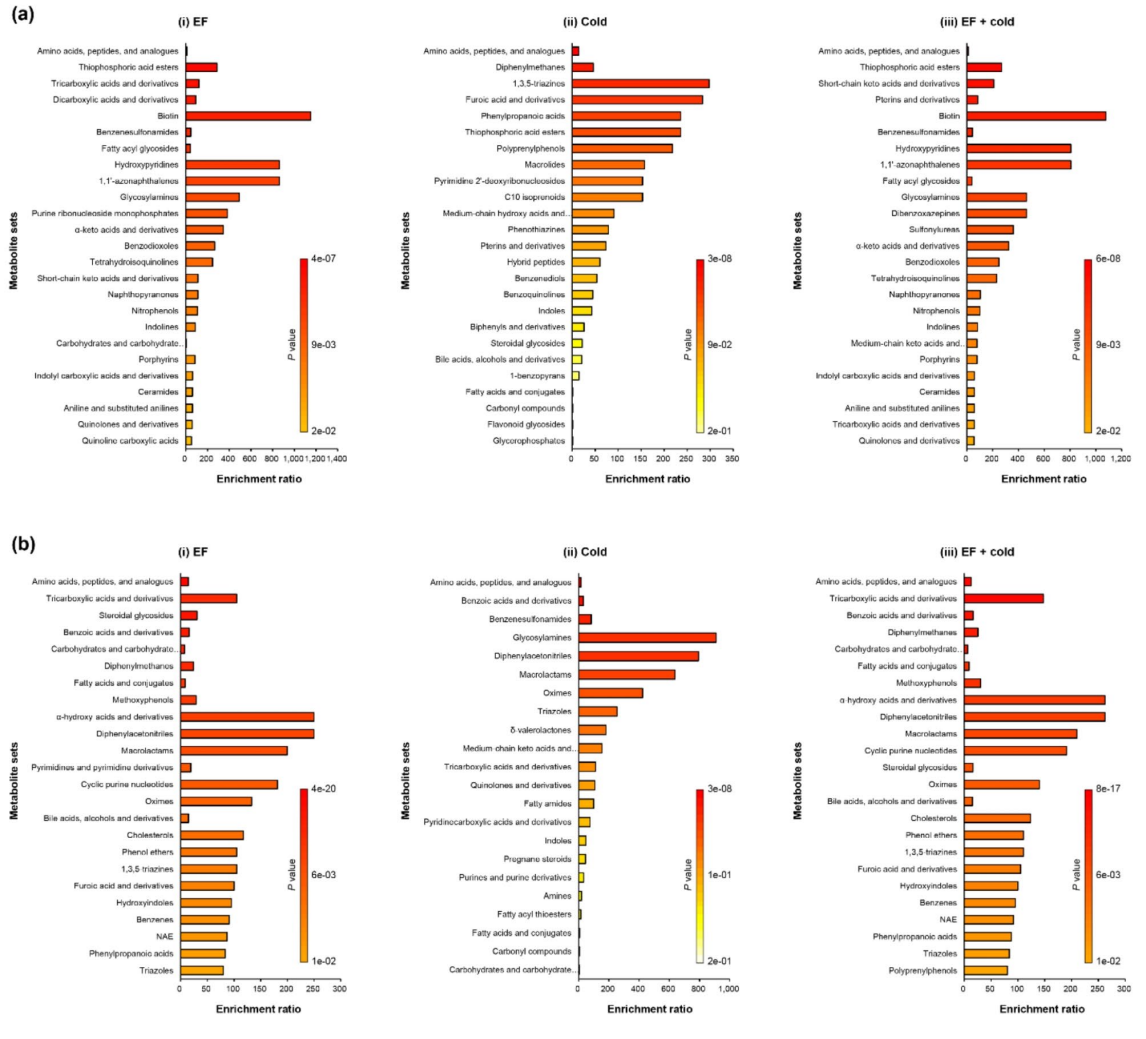
Overview of enriched metabolite sets (top 25).  Metabolite set enrichment analysis of (**a**) upregulated and (**b**) downregulated metabolites. (i) EF, (ii) low temperature, and (iii) combined treatments. The enrichment analysis was performed with (a-i) 179, (b-i) 365, (a-ii) 119, (b-ii) 113, (a-iii) 202, and (b-iii) 335 annotated metabolites. The metabolites were classified into 1250 subchemical class metabolite sets.

Metabolite set enrichment analysis (MSEA) revealed that the classification of metabolites changed in response to the EF, low temperature and combined treatments (Fig. 3). When classified into a set of 1,250 chemical metabolites based on their chemical structures, the most dramatically changed metabolite sets were amino acids and peptides (Fig. 3). Of these, amino acids changed regardless of the specific stress. Compared to the control group, L-arginine was detected, whereas D-ornithine and D-proline were not detected, under all stress conditions. (Table 2). In addition, L-phenylalanine and D-tryptophan showed no changes in metabolism, while L-histidine was upregulated only in the combination treatment group (Table 2).

**Table 2:** Amino acid changes under each stress condition.

Compound	Fold change (raw)				KEGG ID
	[EF]	[Cold]	[EF + Cold]	[Mock]	
L-Arginine	519,758.6	437,847.9	686,403.2	N.D.	C00062
L-Histidine	N.D.	N.D.	122,171.8	58.3	C00135
L-Isoleucine	N.D.	11,798,840.0	N.D.	11,304,809.0	C00407
L-Phenylalanine	20,446,558.0	45,129,472.0	30,474,138.0	42,509,328.0	C00079
D-Ornithine	N.D.	1,260.4	N.D.	29,192.5	C00515
D-Proline	N.D.	N.D.	N.D.	747,820.7	C16435
D-Tryptophan	3,728,519.8	1,591,958.4	3,516,657.2	1,629,028.1	C00525

Purines and pyrimidines, which are heterocyclic aromatic organic compounds, showed interesting metabolite changes under certain stress conditions. Purine ribonucleoside monophosphate was upregulated in the EF alone (Fig. 3a-i), whereas pyrimidine and its derivatives were downregulated (Fig. 3b-i). On the other hand, under low temperature, pyrimidine 2’-deoxyribonucleosides were upregulated (Fig. 3a-ii), whereas purines and their derivatives were downregulated (Fig. 3b-ii). In addition, diphenylmethanes, furoic acids, and phenylpropanoic acids all increased under low temperature (Fig. 3a-ii) but decreased in the EF and combined treatments (Fig. 3b-i and b-iii). Notably, dicarboxylic acids and their derivatives were detected only in the EF (Fig. 3a-i), with phenothiazines and fatty amides likewise only in the low temperature (Fig. 3a-ii and b-ii) and dibenzoxazepines and sulfonylureas only in the combined treatment (Fig. 3b-iii). Overall, the EF alone and the combined treatment showed similar metabolite profiling (Fig. 3a-i and a-iii, 3b-i and b-iii).

### Pathway impact analysis of altered metabolites

**Fig. 4:**
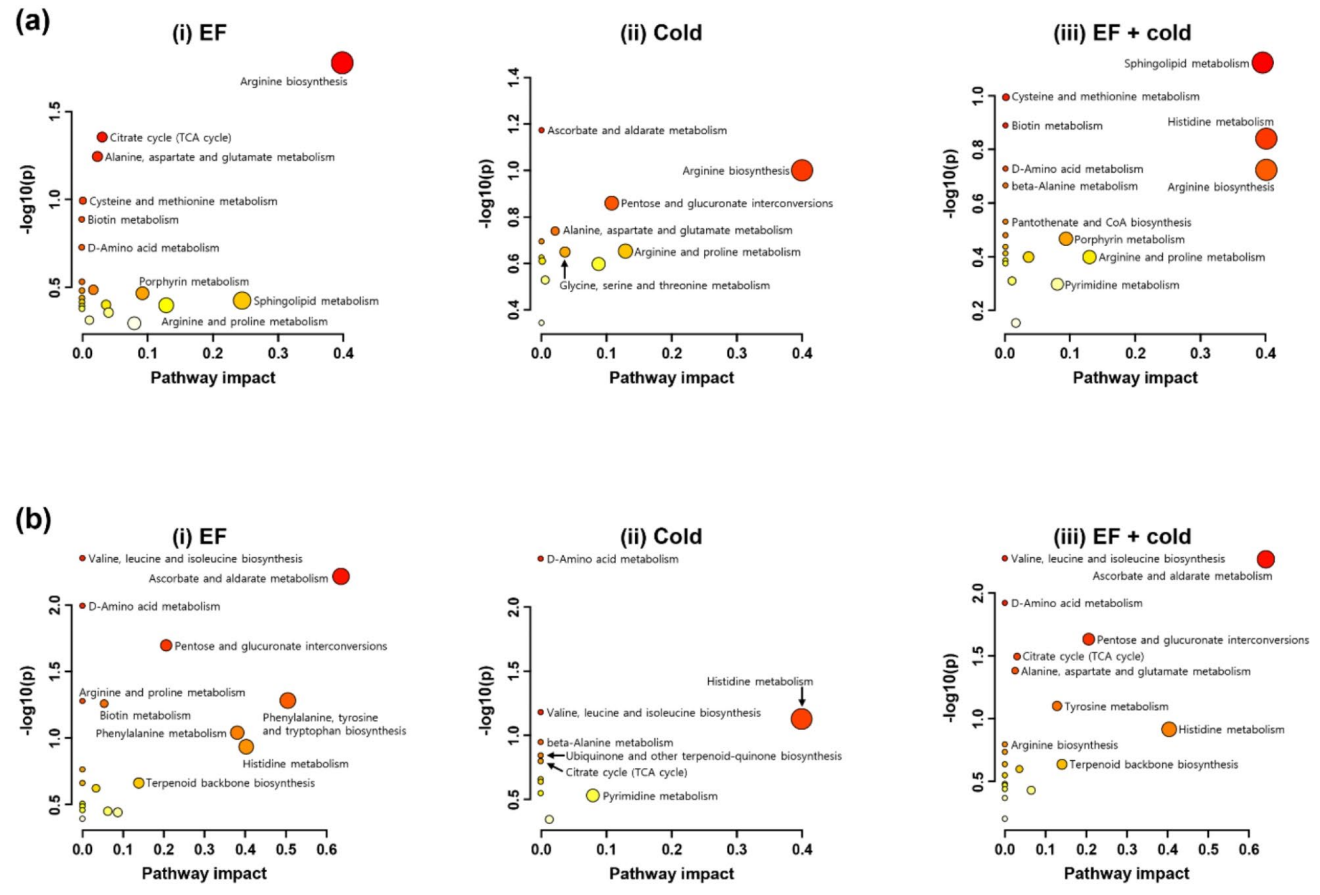
Overview of pathway analysis.  Metabolite pathway analysis of (**a**) upregulated and (**b**) downregulated metabolites. (i) EF, (ii) low temperature, and (iii) combined treatments. The metabolites are presented in Supplementary Data 2.

To investigate the importance of each pathway in the overall metabolic network, metabolites that changed after stress were analyzed by pathway impact score based on the KEGG database (Fig. 4). As expected, amino acid biosynthetic pathways were significantly up- or downregulated under each stress condition. In addition, the TCA cycle was revealed to be one of the major metabolic pathways altered in response to EF fumigation (Fig. 4a-i). These results support the findings of the MSEA that amino acids and the TCA cycle are important under stress conditions. Interestingly, the sphingolipid metabolic pathway was upregulated in the combined treatment, showing strong pathway effect values (Fig. 4a-iii). The EF fumigation and combined treatments showed similar pathway impact (Fig. 4b-i and b-iii), but the low temperature showed a different expression pattern (Fig. 4a-ii and b-ii). Metabolites that have an effect on each metabolic pathway were identified and classified (Supplementary Data 2). Most metabolites functioned in metabolic pathways related to the biosynthesis of amino acids, nucleotides, and cofactors.

NADPH-cytochrome P450 reductase is involved in several biochemical reactions catalyzed by microsomal cytochrome P450 monooxygenases. For example, NADPH-cytochrome P450 reductase functions in the P450 system-mediated detoxification of exogenous xenobiotics and in the regulation of endogenous substrates^27^. Recent studies have shown that RNA interference of NADPH-cytochrome P450 reductase in the rice brown beetle *Nilaparvata lugens* and the small brown planthopper *Laodelphax striatellus*increases susceptibility to insecticides^27,28^. In this process, NADPH-cytochrome P450 reductase transfers electrons derived from the hydride ion of NADPH to cytochrome P450 via flavin mononucleotide (FMN) and FAD^29^. In this study, FMN was significantly less abundant in the EF alone and combined treatments than in the control group (Supplementary Data 2). These results suggest that the inhibition of FMN biosynthesis by EF fumigation reduces NADPH-cytochrome P450 reductase activity, resulting in decreased survival in *D. suzukii*.

Insecticides cause oxidative stress in cells and generate reactive oxygen species (ROS) free radicals^30^. Glutathione (GSH) acts as a cofactor to scavenge toxic radicals. During redox stress, GSH levels decrease, and glutathione disulfide (GSSG) levels increase in the presence of glutathione peroxidase^31–33^. In this study, EF and low temperatures led to high relative changes in GSSG metabolites (Supplementary Data 2). These results suggest that insect immune responses are induced by EF fumigation and by low temperature because insect glutathione-S-transferases (GSTs) play a role in protecting the host from oxidative stress caused by insecticide exposure^34^.

Although they were not identified as major substances in the pathway analysis based on the KEGG database, several substances of importance to insect physiology were detected by metabolomics (Supplementary Data 3). Despite the importance of vitamin D_3_, its metabolic mechanisms and physiological roles in invertebrate insects are still unknown. 7-dehydrocholesterol (7DHC) is known as the precursor of the 20-hydroxyecdysone hormone and vitamin D_3_in insects^35,36^. In this study, 11 vitamin D_3_-related metabolites were identified, though none were detected in the EF or combined treatments (Supplementary Data 3). These results suggest that EF caused dysfunction of the precursor 7DHC and consequently inhibited the biosynthesis of vitamin D_3_. Considering the synthetic pathway of 7DHC, dysfunction of the precursor 7DHC would also have inhibited the effect of 20-hydroxyecdysone on pupal *D. suzukii*, resulting in an insecticidal effect.

Interestingly, metabolites related to prostaglandin biosynthesis, an insect immune system, were significantly altered (Supplementary Data 3). Prostaglandin F_2α_ and thromboxane A2 were not detected in the EF alone, suggesting that EF fumigation completely suppressed the insect immune response.

### Lipid classes and statistical analysis changes under stress conditions

**Fig. 5:**
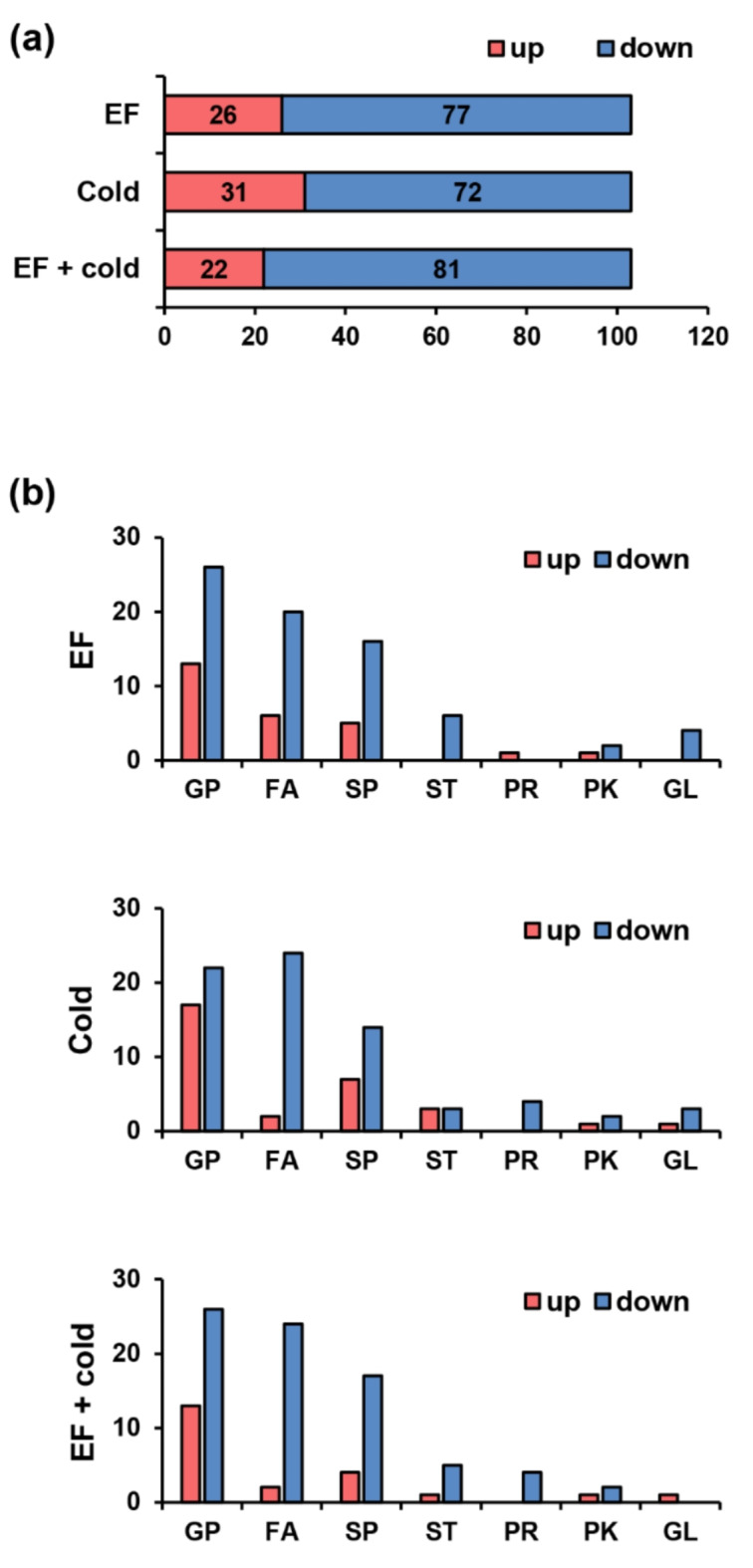
Lipidomic profiling altered by exposure to the fumigant EF and low temperature.  (**a**) The number of lipids showing relative increases and decreases in D. suzukii after stress. (**b**) The number of lipids showing relative increases and decreases according to lipid class. GP: glycerophospholipids; FA: fatty acids; SP: sphingolipids; ST: sterol lipids; PR: prenol lipids; PK: polyketides; GL: glycerolipids. The lipid data are presented in Supplementary Data 4.

Since sphingolipid metabolism significantly changed in response to the combined treatment according to the pathway impact analysis, the effect of each stress on the lipid pathway in *D. suzukii* was also analyzed. Lipid profiles in response to stress were filtered by multivariate statistical analysis (Supplementary Data 4). A total of 103 lipids were identified through filters and annotations, and many lipids showed a downward trend compared to the control (Fig. 5a). The majority of lipid classes, including glycerophospholipids (GPs), fatty acids (FAs), and sphingolipids (SPs), changed, while sterol lipids (STs), prenol lipids (PRs), polyketides (PKs), and glycerolipids (GLs) showed minor changes (Fig. 5b). Additionally, when MSEA was performed on the distribution and metabolic pathways of lipid subclasses changed by stress, metabolites involved in sphingolipid and ceramide biosynthesis were significantly altered both numerically and statistically (Supplementary Fig. S1). These results indicate that cold acclimation causes lipid and metabolic changes in *D. suzukii*^37^.

### Pathway analysis of sphingolipid metabolism

**Fig. 6:**
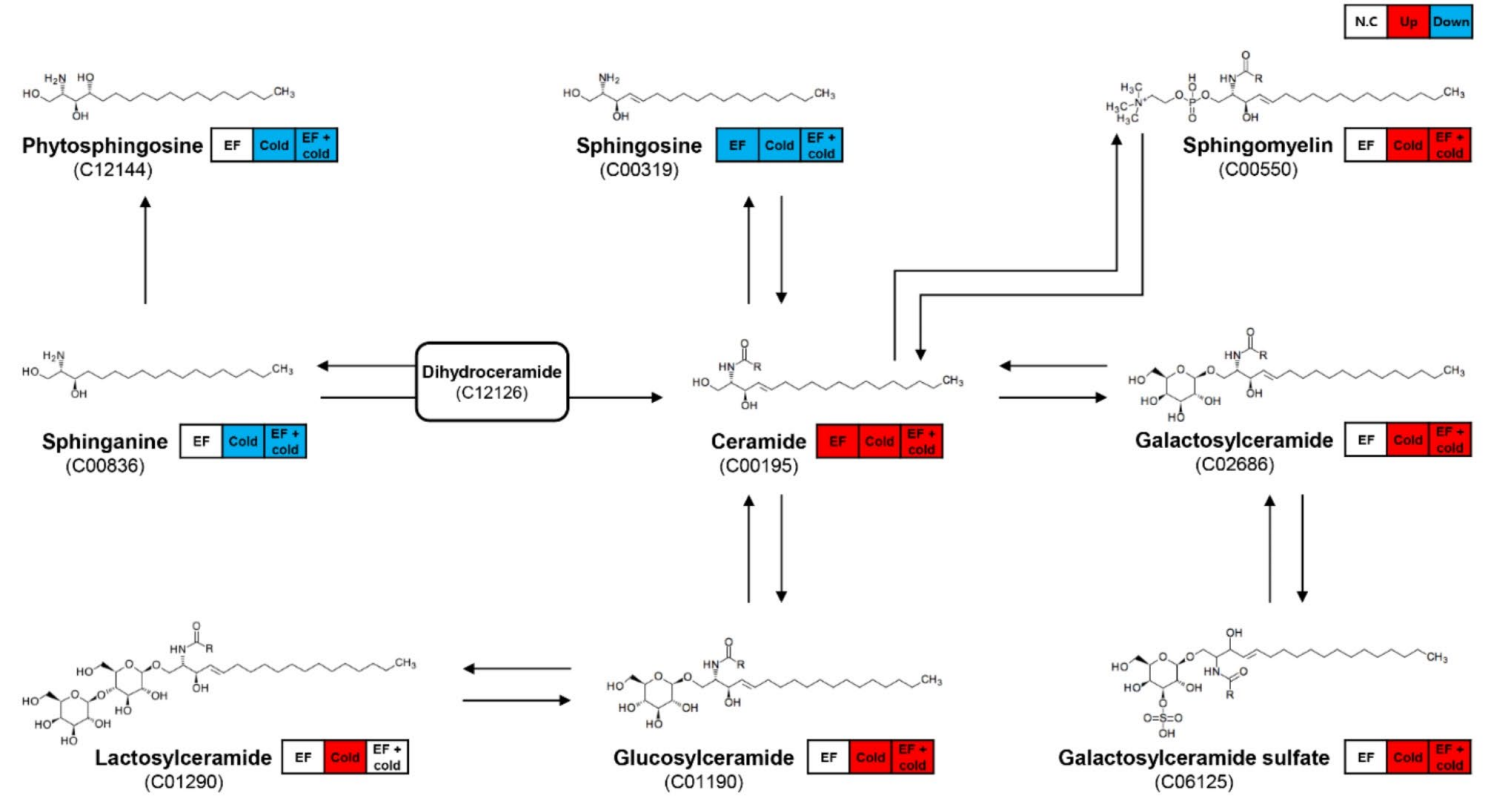
Pathway analysis of sphingolipid metabolism. Up- and downregulated lipids are indicated by red and blue lines, respectively. The numbers represent the KEGG IDs.

Sphingolipids are components of lipid rafts that are involved in cell membrane receptor and signal transduction^38–40^. Since a recent study showed that low temperature causes changes in the structure and profile of lipid rafts, the metabolic processes of sphingolipids were investigated to determine their relationships with stress^41^. Sphingosine, sphinganine, and phytosphingosine were downregulated under stress conditions, while ceramide-related metabolites were upregulated (Fig. 6). These patterns did not differ in response to stress. Our previous study revealed EF insecticide susceptibility at each developmental stage of *D. suzukii*and revealed synergistic effects of fumigants and low temperature^1^. Combining the in vivo insecticidal effect and in vitro lipidomic data, it can be suggested that low temperature had an additional effect on the immune response by adjusting the lipids of *D. suzukii*.

## Conclusion

There are many studies on eco-friendly control agents using various fumigants or low temperatures in the *D. suzukii* model. However, physiological changes in *D. suzukii* that are induced or reduced by a combination of fumigants and low temperatures have not been elucidated at the molecular level. Therefore, considering the biochemical mechanisms by which EFs inhibit cytochrome oxidase activity in the electron transport chain of intracellular respiration, studies on metabolic changes are needed to understand any potential synergistic effects. In this study, we investigated how metabolites significantly altered by fumigants and low temperatures correlate with physiological changes in insects.

Most of the metabolites in the *D. suzukii* metabolic pathway are associated with the biosynthesis of amino acids, nucleotides and cofactors, and the relative changes in the metabolic pathway in the fumigant-treated group were fewer than those in the control and the low temperature. These specific cofactors are involved in the detoxification and immune response of *D. suzukii*, providing evidence for the mechanism of EF’s action. Since amino acids and nucleotides are basic physiological materials, alterations in these metabolites could be important indicators for tracking physiological changes in *D. suzukii*. Therefore, this study provides useful data for the development of biomarkers for fumigation or low temperature during the quarantine process.

## Electronic supplementary material

Below is the link to the electronic supplementary material.

Supplementary Material 1

Supplementary Material 2

Supplementary Material 3

Supplementary Material 4

Supplementary Material 5

## Data Availability

All data generated or analysed during this study are included in this published article [and its supplementary information files].
