# Supplementary material for: Metabolite changes by combined treatment, ethyl formate and low temperature, in Drosophila suzukii
Source: Sci Rep. 2024 Oct 29;14:25948. doi: 10.1038/s41598-024-77436-0 (PMC11522300; doi:10.1038/s41598-024-77436-0)

Supplementary Figure 1.

Enriched lipids sets in (i) EF, (ii) low temperature and (iii) combined treatment. The lipids were presented in Supplementary Data 4.

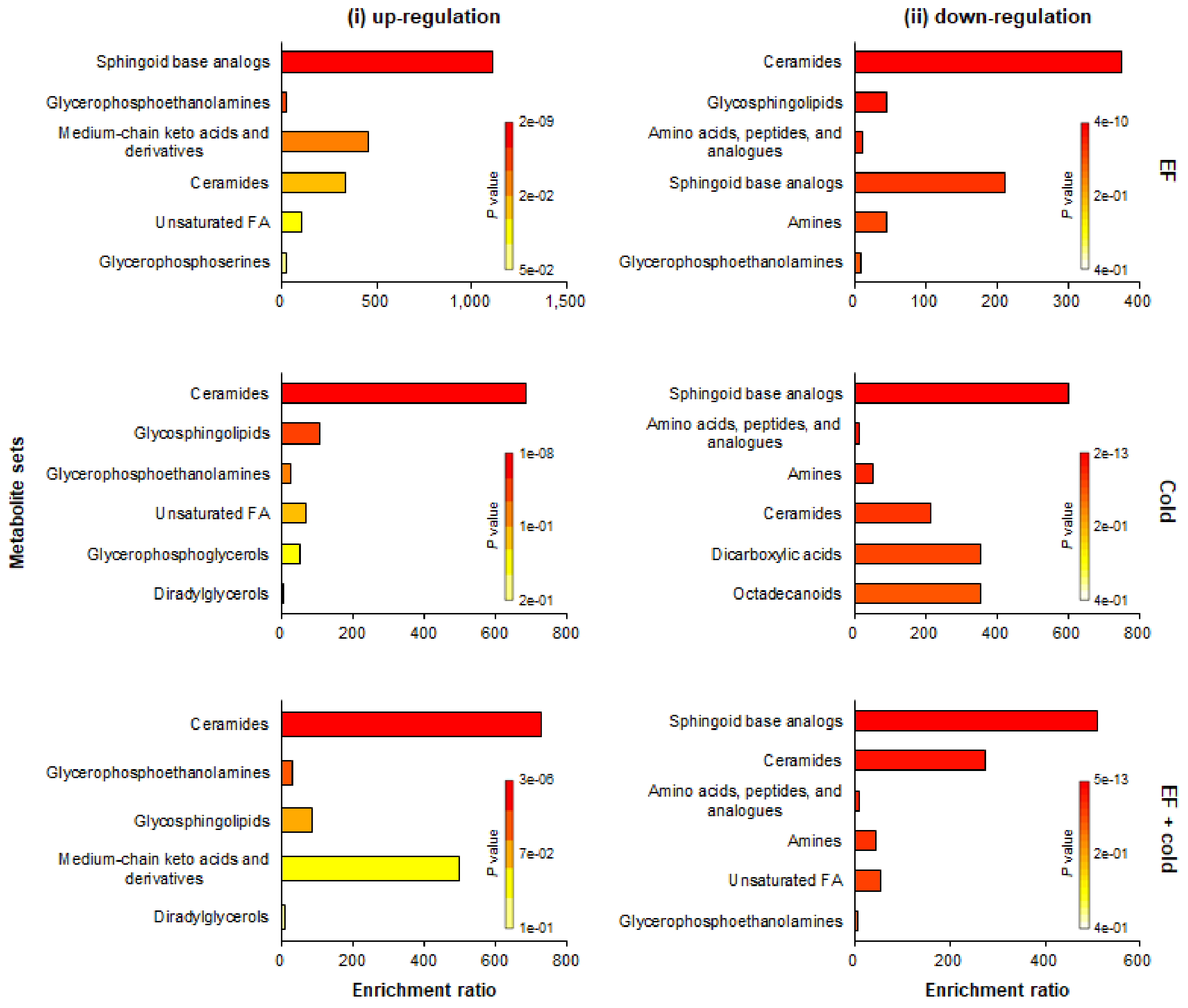

Supplement: Supplementary file 5 — Supplementary Material 5 [file 41598_2024_77436_MOESM5_ESM.pdf]
